# Developments in MRI radiomics research for vascular cognitive impairment

**DOI:** 10.1186/s13244-025-02026-1

**Published:** 2025-07-01

**Authors:** Xuezhi Chen, Xianting Luo, Liang Chen, Hao Liu, Xiaoping Yin, Zhiying Chen

**Affiliations:** 1 University of Shanghai for Science and Technology, Shanghai, China; 2 Department of Neurology, Affiliated Hospital of Jiujiang University, Jiujiang, China; 3 Department of Imaging, Affiliated Hospital of Jiujiang University, Jiujiang, China

**Keywords:** Vascular cognitive impairment, Radiomics, Magnetic resonance imaging, White matter lesions, Artificial intelligence

## Abstract

**Abstract:**

Vascular cognitive impairment (VCI) is an umbrella term for diseases associated with cognitive decline induced by substantive brain damage following pathological changes in the cerebrovascular system. The primary clinical manifestations include behavioral abnormalities and diminished learning and memory cognitive functions. If the location and extent of brain injury are not identified early and therapeutic interventions are not promptly administered, it may lead to irreversible cognitive impairment. Therefore, the early diagnosis of VCI is crucial for its prevention and treatment. Prior to the onset of cognitive impairment in VCI, magnetic resonance imaging (MRI) radiomics can be utilized for early assessment and diagnosis, thereby guiding clinicians in providing precise treatment for patients, which holds significant potential for development. This article reviews the classification of VCI, the concept of radiomics, the application of MRI radiomics in VCI, and the limitations of radiomics in the context of advancements in its application within the central nervous system.

**Critical relevance statement:**

This article explores how MRI radiomics can be used to detect VCI early, enhancing clinical radiology practice by offering a reliable method for prediction, diagnosis, and identification, which also promotes standardization in research and integration of disciplines.

**Key Points:**

- MRI radiomics can predict VCI early.
- MRI radiomics can diagnose VCI.
- MRI radiomics distinguishes VCI from Alzheimer’s disease.

**Graphical Abstract:**

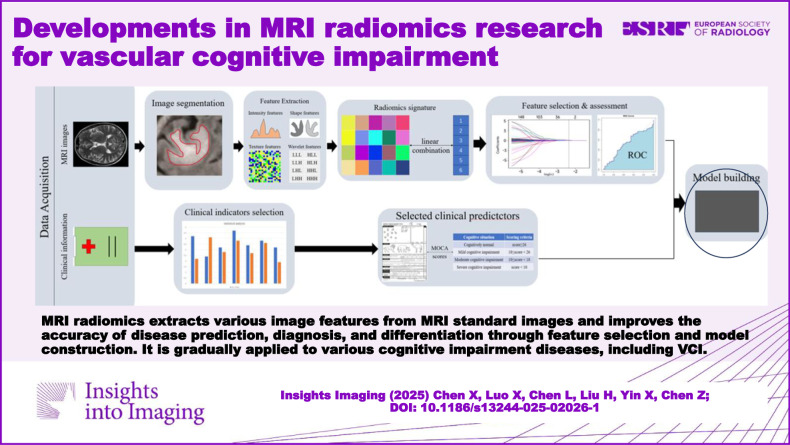

## Introduction

Vascular cognitive impairment (VCI) is the second most prevalent form of dementia, surpassed only by Alzheimer’s disease (AD) [1]. It encompasses a range of disorders linked to cognitive decline that arise from pathological alterations in the vascular system. The condition typically progresses through three clinical stages, beginning with subjective cognitive decline, advancing to mild cognitive impairment (MCI), and ultimately evolving into dementia [2]. Globally, nearly all countries are experiencing a rapid increase in the proportion of the population aged 60 and over, due to declining birth rates and rising life expectancy. According to United Nations statistics from 2019, the global population aged 65 and over exceeds 703 million, accounting for 9% of the world’s total population. It is projected that this proportion will rise from 10% in 2022 to 16% by 2050, representing approximately 1.5 billion elderly individuals [3]. Two-thirds of individuals aged 60 and above, due to risk factors for stroke such as hypertension, face a significantly increased risk of developing VCI [4]. The 2018 World Alzheimer’s Report stated that globally, 20 million people suffer from dementia caused by vascular diseases. Particularly in low- and middle-income countries, where cardiovascular diseases are not adequately controlled, the number of patients is expected to continue to rise significantly in the coming decades. The escalating number of dementia patients has resulted in substantial costs worldwide, and with the advancement of medical standards across nations, it is highly probable that expenditures on dementia will continue to increase in the coming years [5]. Moreover, VCI significantly increases the likelihood of patients developing depression and experiencing a decline in quality of life [6]. Under the current circumstances, the clinical assessment of the severity of vascular brain injury through neuroimaging is often imprecise, and the relationship between vascular lesion burden and cognitive function exhibits significant inter-subject variability [7]. Moreover, there is a paucity of pharmacological or surgical interventions available in the clinical setting to ameliorate the condition of patients with VCI. The primary approach to VCI prevention involves the management of vascular risk factors or conditions such as hypertension and diabetes, which are known to predispose individuals to VCI [2]. Consequently, there is a pressing need to identify a method that can effectively diagnose and predict the onset of VCI. Such a method would be of paramount importance in enhancing the quality of life for patients with cerebrovascular diseases and in alleviating the fiscal strain imposed by the costs associated with VCI on nations. Currently, magnetic resonance imaging (MRI) is routinely employed in research related to cerebrovascular diseases; however, it falls short in facilitating the early diagnosis of VCI and in forecasting the progression of dementia in patients. The emerging field of radiomics offers a novel solution to this challenge by enabling the quantitative extraction of diverse imaging features from standard MRI images. Through the process of feature selection followed by model construction, radiomics provides a more precise means of predicting, diagnosing, and differentiating diseases, thereby opening new avenues to address this issue. Historically, radiomics has predominantly been applied in the study of oncological pathologies [8]. In recent times, however, its application has expanded to encompass the prediction, diagnosis, and differentiation of VCI. In light of this, the present article aims to provide a comprehensive review of the classification of VCI, the conceptual framework of radiomics, the application of MRI-based radiomics in the context of VCI, and the inherent limitations of radiomics.

## Classification of VCI

VCI is primarily caused by risk factors affecting the cerebrovascular system, such as hyperlipidemia, hypertension, and diabetes, which are prone to inducing cerebrovascular diseases, leading to cognitive impairment in individuals. VCI can be categorized into three subtypes based on the severity of cognitive impairment and the type of pathology: vascular cognitive impairment no dementia (VCIND), vascular dementia (VD), and mixed dementia [9]. By classifying VCI, doctors can more accurately develop personalized treatment plans and assess the prognosis for patients in different subgroups. Among them, VCIND is a condition characterized by cognitive impairment, referring to damage in one or more cognitive domains that does not affect instrumental activities of daily living (IADL) or activities of daily living (ADL). This cognitive impairment is caused by vascular damage in the brain but does not yet meet the criteria for dementia [10]. VCIND is a milder form of VCI compared to VD [11]. Although VCIND does not yet meet the criteria for dementia, individuals with VCIND are at a higher risk of developing dementia in the future [12]. VD refers to a severe cognitive dysfunction syndrome caused by stroke or cerebrovascular diseases [13], characterized by significant impairment in one or more cognitive domains that affects IADL or ADL. It is the second leading cause of dementia, following AD, accounting for 30% of dementia cases in Asia [14]. The main manifestations include declines in memory, calculation ability, language, attention, and executive function [15]. The cause of VD is multifactorial, leading to neurovascular damage, which further results in cognitive dysfunction. Its primary pathogenic factors include oxidative stress, neuroinflammatory responses, blood-brain barrier disruption, and neurotoxicity [16]. Mixed dementia typically manifests with multiple underlying pathologies, the most common form being a combination of AD and VD. In the past, the definitive diagnostic method was autopsy [17]. Additionally, Chinese scholars have found through statistics that the incidence of cognitive impairment is 80.97% in stroke patients, 48.91% in VCIND, and 32.05% in VD [18]. Therefore, the prevention and treatment of VCI are of utmost urgency. Hence, there is a clinical demand for a method that can simply and accurately predict, diagnose, and differentiate VCI. The emergence of radiomics provides such a method. It allows for radiomics analysis of patients after they have developed vascular diseases, predicting and diagnosing whether they will suffer from VCI.

## The concept of MRI radiomics

**Fig. 1:**
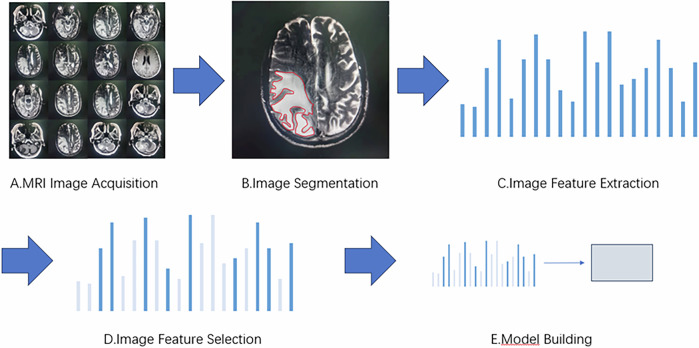
Imaging radiomics flowchart. **A** MRI image acquisition refers to obtaining standard MRI image data, which is the foundation of radiomics. These data mainly come from medical institutions or public datasets. **B** Image segmentation is the process of segmenting an image into specific brain regions with biological significance or extracting regions of interest. **C** Feature extraction is the step of identifying quantitative features from segmented images that are helpful for disease diagnosis and analysis. **D** To avoid overfitting and improve accuracy, it is necessary to select the most relevant features to the disease state from a large number of extracted features before modeling. **E** The ultimate goal of radiomics is to establish a model for diagnosis, classification, and prediction

Lambin first proposed the concept of radiomics in 2012 [19]. Radiomics is the high-throughput extraction of quantitative imaging features from medical images (such as CT and MRI images) and the application of these data to clinical decision-support systems to improve the accuracy of diagnosis, prediction, and prognosis of certain diseases [20]. It has been validated in various diseases, including lung cancer [21], nasopharyngeal carcinoma [22], breast cancer [23], colorectal cancer [24], Crohn’s disease and intestinal fibrosis [25], and so on. MRI radiomics (Fig. 1) is a branch of radiomics that includes MRI image acquisition, image segmentation, feature extraction, feature selection, and model building [26].

### Image acquisition and segmentation

Image acquisition serves as the foundation for radiomics analysis, typically obtained through local medical institutions [27] or regional picture archiving and communication systems to access standardized MRI image data. A series of preprocessing steps is then applied to the MRI images, such as histogram equalization and image registration methods [28]. The commonly used sequences in current research on cognitive impairment include T1WI, T2WI, FLAIR, DWI, and DTI sequences [29]. Image segmentation is the process of dividing images into regions of interest (ROI) with biological significance. It generally involves three methods: manual segmentation, semi-automatic segmentation, and automatic segmentation [30]. Manual segmentation is highly accurate and is widely regarded as the reference standard when performed by experienced observers [31]. In recent years, with the rapid development of computer technology, semi-automatic segmentation and automatic segmentation have also seen significant progress [32]. Semi-automatic segmentation can greatly reduce the segmentation workload for humans, but still requires manual correction and verification of accuracy. Automatic segmentation can eliminate the errors caused by human subjectivity, significantly improve efficiency and reproducibility, and there are many open-source software programs available for segmentation. However, the schemes and standards for automatic segmentation have not yet been unified. Currently, commonly used open-source software includes ITK-SNAP [33] and 3D Slicer [34] among others.

### Image feature extraction and feature selection

**Fig. 2:**
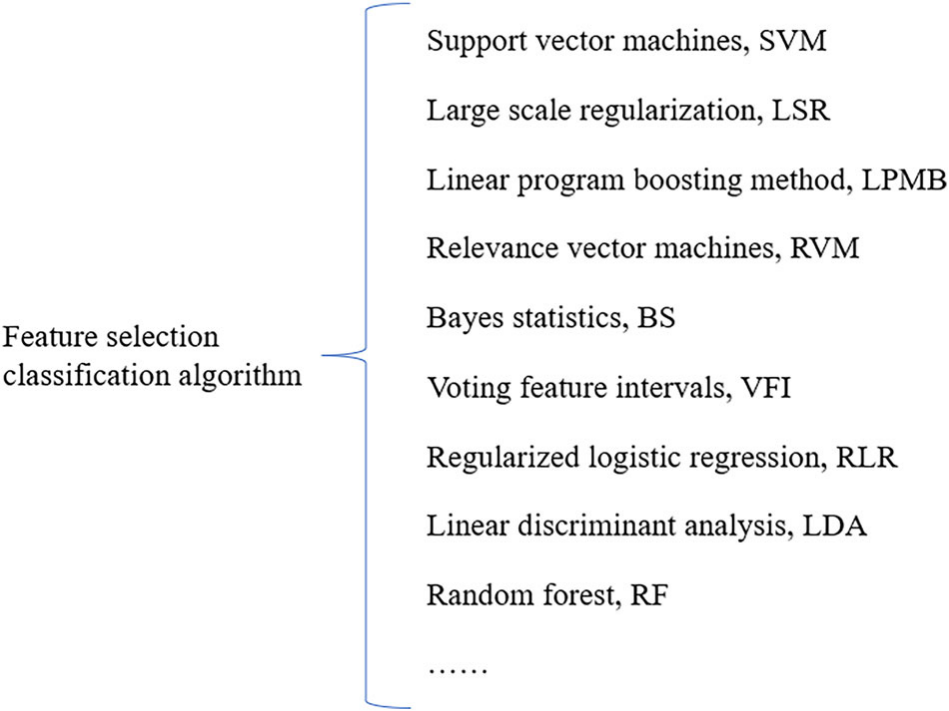
Feature selection classification algorithm

Image feature extraction is the next step after image segmentation and is also a core step in radiomics, which involves identifying quantifiable features that aid in disease diagnosis and analysis from the segmented images. Commonly used feature extraction tools in radiomics currently include MATLAB [35], MaZda [36], and pyradiomics [37], among others. After feature extraction, a large array of features is usually obtained. To avoid the “curse of dimensionality” due to high complexity, feature selection and dimensionality reduction are needed. This eliminates irrelevant and interfering features, thereby enhancing the interpretability and generalizability of radiomic models and reducing computational complexity and overfitting risks. Feature selection methods include the following three types: filtering, embedding, and wrapping. Filter methods rank features based on relevance or a specific characteristic, then set a threshold or select the top-ranked ones. Embedded methods directly select features via a trained model that assigns weight coefficients to each feature, using these coefficients to pick optimal features. Wrapper methods evaluate feature (subsets) quality by model performance, iteratively retaining or removing features [38]. Commonly used classification algorithms (Fig. 2) include support vector machines (SVM), relevance vector machines, random forests (RF), and so on.

### Model building

After feature selection of the images, various modeling methods can be selected according to the needs of the study, such as prediction, summarization, classification, etc., including SVM, RF, convolutional neural networks (CNN) [39], and so on. The generalization and performance of the model are usually assessed using methods such as the area under the curve (AUC) and receiver operating characteristic (ROC) for external or cross-validation [40], and the model parameters are verified for correctness through clinical analysis.

## Application of MRI radiomics in VCI

**Fig. 3:**
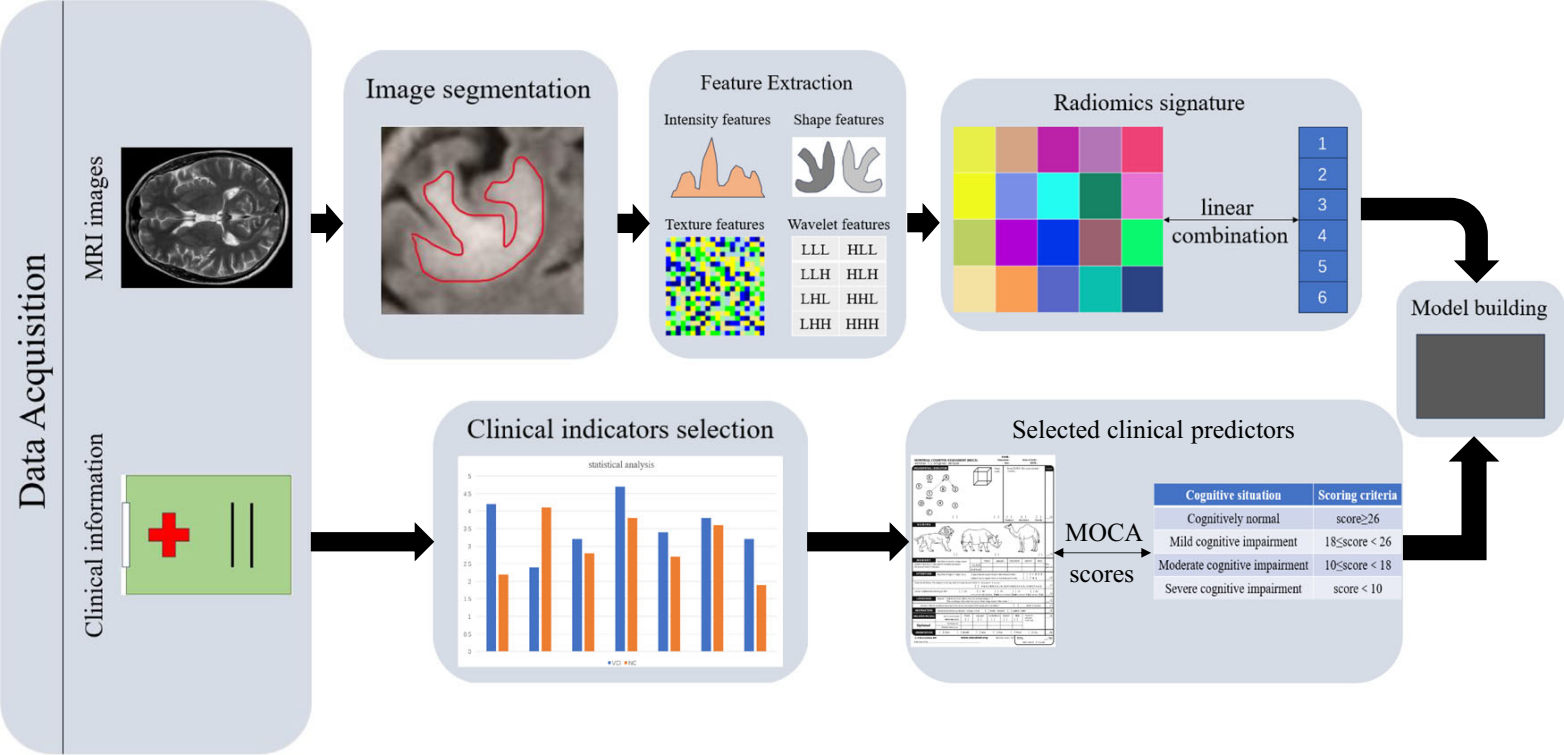
MRI imaging radiomics flowchart of VCI

Radiomics in MRI extracts various image features from standard MRI images and improves the accuracy of disease prediction, diagnosis, and differentiation through feature selection and model construction [41]. It is gradually being applied to various cognitive impairment diseases [42], including VCI (Fig. 3).

This study followed the PRISMA guidelines and conducted a comprehensive search in PubMed, Web of Science, and Cochrane Library databases, with a focus on articles related to the application of radiomics in VCI. The following keywords were used: “radiomics”, “artificial intelligence”, “machine learning”, “deep learning” and “vascular cognitive impairment”, “vascular dementia”, “post stroke dementia”, “subcortical ischemic vascular dementia”, “mixed dementia”. Specific search strings were constructed for each library to ensure thorough retrieval.

**Fig. 4:**
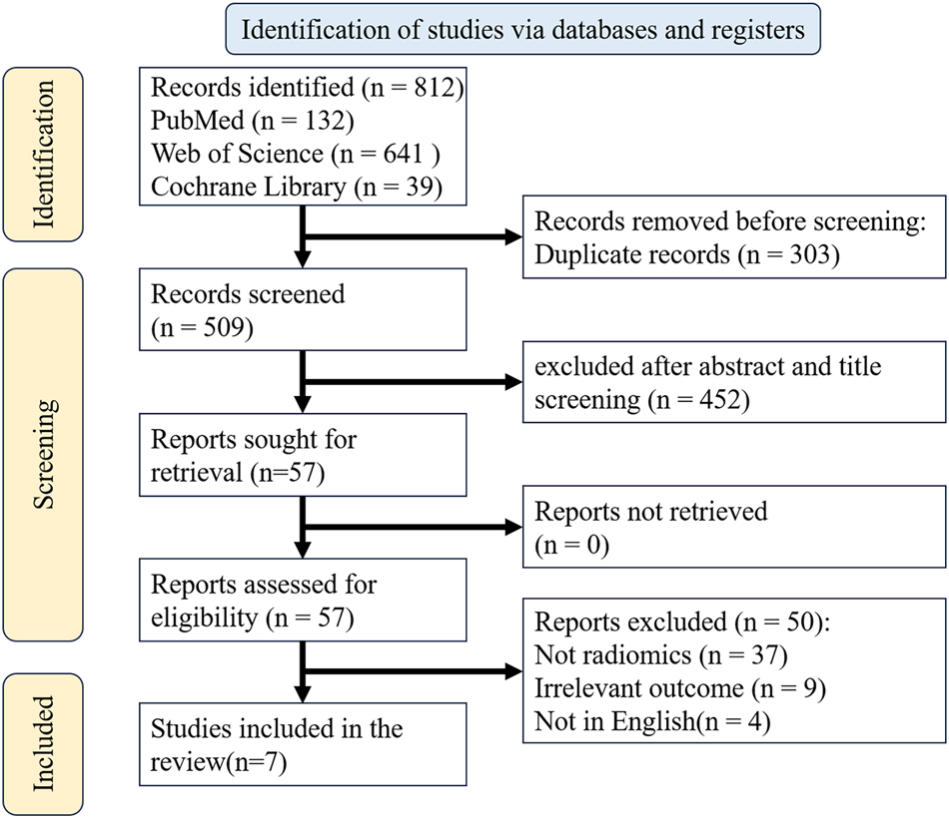
The process of study inclusion based on PRISMA methodology

We included original research articles involving human subjects in our analysis, which were written and published in English from January 1, 2015, to December 31, 2024. After initial retrieval, the eligibility of duplicate items is screened and evaluated based on their correlation with radiomics and VCI. This stage involves carefully reviewing the title and abstract, followed by full-text screening of the selected articles. The detailed process of research inclusion can be summarized as the following flowchart based on the PRISMA method (Fig. 4). Therefore, preliminary searches were conducted in three databases, resulting in 812 studies, of which 303 studies were screened after removing duplicates. After screening 452 studies through abstracts and titles, 57 were retrieved, and 50 were excluded. A total of 7 studies were included in the review.

### Application of MRI radiomics in VCI prediction

**Fig. 5:**
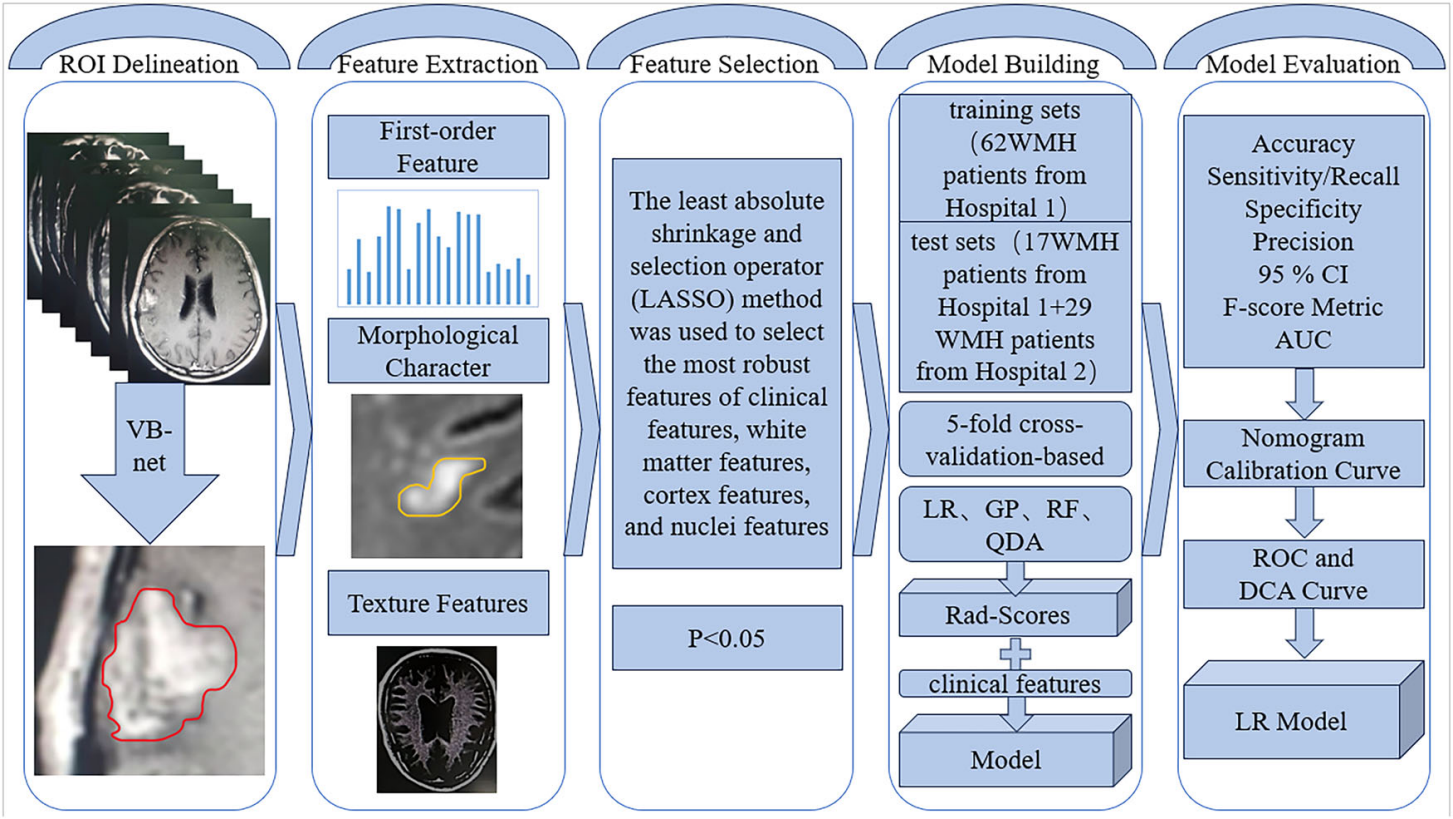
The research process of Feng et al

Conventional MRI examinations find it difficult to predict the progression of VCI. Therefore, some scholars have begun to use MRI radiomics to study the prediction of VCI. A prospective screening study has shown that seven types of pathology, including large infarcts, lacunar infarcts, microinfarcts, myelin loss, small vessel disease, cerebral amyloid angiopathy (CAA), and perivascular space expansion, can predict cognitive impairment [43]. Shahram Oveisgharan et al [17] analyzed autopsy data from 1767 subjects between May 2021 and July 2022 and concluded that pure VCI, defined as cognitive impairment caused by pathological cerebrovascular changes, is not uncommon among these subjects, with white matter infarction being the primary cerebrovascular pathology leading to cognitive decline or impairment. Cerebral small vessel disease is the main cause of VCI [44]. Insufficient perfusion caused by small vessel disease or chronic cerebral ischemia can lead to white matter lesions [45], which are associated with cognitive decline and dementia [44]. Feng et al [46] utilized radiomics and deep learning to develop a model for detecting cognitive impairment in patients with white matter hyperintensities (WMH) (Fig. 5). They randomly divided 79 WMH patients from Hospital 1 into a training set of 62 cases and a testing set of 17 cases, and additionally included 29 patients from Hospital 2 in an independent testing set. They used VB.NET for automatic identification and segmentation of WMH and extracted radiomics features from the nuclei, cortex, and white matter. Four machine learning classifiers were trained on the training set and validated on the testing set to detect cognitive impairment. They evaluated and compared the performance of the models and conducted a causal analysis between cognitive impairment and changes in nuclei, cortex, and white matter. They found that the logistic regression (LR) model based on white matter features showed the highest performance among these models, with an AUC of 0.819 in the external test dataset. The analysis indicated that changes in nuclei, cortex, white matter, age, and education level are pathogenic factors for cognitive impairment. Therefore, the LR model based on white matter features and radiomics has high accuracy in detecting cognitive impairment in WMH patients. The study combined deep learning and radiomics, using multicenter data and causal analysis to demonstrate the potential causal relationship between cortical, white matter, and nuclei changes and cognitive impairment. It provides an effective tool for early diagnosis and an important reference and foundation for future related research.

Some scholars have also directly used radiomics analysis of arteries to predict the development of VCI. Studies have shown that a decrease in the length of the lenticulostriate arteries (LSA) increases the likelihood of an increased number of lacunar infarcts in the basal ganglia [47]. Zhou et al [48] investigated the correlation between LSA and cognitive impairment using radiomics and clinical features. They retrospectively included 102 patients with a mean age of 62.5 ± 10.3 years, divided into 58 cases of MCI and 44 cases of moderate to severe cognitive impairment (MSCI). The MRI images of these 102 patients were preprocessed with *Z*-score normalization, and ROI were manually delineated. Radiomics features were extracted using PyRadiomics. Feature selection was performed using methods such as least absolute shrinkage and selection operator (LASSO), maximum relevance and minimum redundancy, and univariate analysis. A multivariate LR model was used for construction, and the model was assessed using calibration curves (CC), ROC, and decision curve analysis (DCA). In the training dataset of 71 patients (including 44 MCI patients) and the testing dataset of 31 patients (including 17 MCI patients), the combined AUC was 0.88 [95% CI 0.78, 0.97] in the training set and 0.76 [95% CI 0.6, 0.93] in the testing set, which was superior to the radiomics and clinical models alone. The DCA results indicated that the combined model had the highest net benefit compared to the radiomics and clinical models. This study demonstrated that the combined model integrating clinical and radiomics features of LSA can effectively predict MCI, and LSA vascular parameters can serve as imaging biomarkers for cognitive impairment. It highlights the potential of radiomics technology in VCI research and provides important references and foundations for future related studies.

Some scholars have also explored VCI indirectly through radiomics studies of amyloid proteins. This is because amyloid β-protein (Aβ) is present within the walls of blood vessels and, in most cases, appears as amyloid plaques. This pathological change is referred to as CAA [49]. Predicting amyloid positivity can be used to detect CAA, thereby predicting VCI. Kim et al [50] used structural MRI and radiomics methods to predict amyloid positivity. They extracted radiomics features composed of texture features and histograms from MRI images of 440 MCI patients, which included T1, T2 FLAIR, and DTI sequences. These radiomics features could be used either alone or in combination with baseline non-imaging predictors (such as ApoE genotype, age, and gender) to predict amyloid positivity, with feature selection and prediction performed using regularized regression methods. The performance of the baseline non-imaging model was at a fair level (AUC = 0.71). Radiomics models based on individual MRI sequences (T1 and T2 FLAIR) also predicted amyloid positivity well, with test AUCs of 0.71–0.74 and validation AUCs of 0.68–0.70. When T1 and T2 FLAIR radiomics features were combined, the test AUC was 0.75 and the validation AUC was 0.72, with *p* < 0.001 compared to the baseline model. When baseline features were combined with the T1 and T2 FLAIR radiomics model, the test AUC was 0.79 and the validation AUC was 0.76, showing the best performance and significantly outperforming the baseline model (*p* < 0.001) and the T1 + T2 FLAIR radiomics model (*p* < 0.001). The study concluded that radiomics features have predictive value for amyloid positivity and can enhance prediction performance when combined with other predictive functions, thereby enabling the prediction of VCI. The study demonstrated that a combined model of radiomics features from T1 and T2 FLAIR sequences and clinical variables has the best potential value in predicting amyloid positivity in MCI patients. This approach can reduce the screening failure rate in clinical trials and save resources.

From the studies mentioned above, it is evident that radiomics can be used to analyze white matter changes caused by small vessel disease or cerebral ischemia, which are associated with cognitive decline, thereby indirectly predicting VCI. Additionally, radiomics combined with clinical features of LSA can be used to predict MCI, enabling rapid diagnosis of vascular MCI and facilitating prompt treatment. Furthermore, MRI radiomics can be employed to analyze amyloid plaque deposition within blood vessels, aiding in the assessment of CAA and predicting the progression of VCI for early detection. Therefore, researchers can use radiomics to directly or indirectly predict VCI through various influencing factors.

### Application of MRI radiomics in VCI diagnosis

The application of MRI radiomics in VCI not only includes the prediction of VCI but also research on the diagnosis of VCI. Traditionally, the diagnosis of VCI has relied on clinical and neuropsychological assessments, as well as neuroimaging evaluations and analyses [51]. In the field of radiomics, Xue et al [52] explored the impact of MRI image features based on radiomics and deep learning algorithms on patients with cerebrovascular disease. They selected 80 patients with acute cerebrovascular disease as research subjects, who were divided into a VCI group of 34 cases and a non-VCI group of 46 cases based on the presence or absence of VCI. They proposed a new multi-modal CNN image segmentation algorithm based on CNN and applied it to the segmentation of MRI images of VCI patients. The segmentation results were compared with those of the fully convolutional network and CNN algorithms. The Dice coefficient of the multi-modal CNN algorithm was 0.78 ± 0.24, the accuracy was 0.81 ± 0.28, and the recall rate was 0.88 ± 0.32, all of which were significantly higher than the other two algorithms (*p* < 0.05). In neurological assessments, the VCI group had TMT-a and TMT-b scores of 221.7 and 385.9, respectively, which were significantly higher than those of the non-VCI group (*p* < 0.05). The MMSE and MoCA scores of VCI patients were 15.4 and 14.6 ± 5.31, respectively, which were significantly lower than those of the non-VCI group (*p* < 0.05). The study showed that the FA and MD values of nerve function-related fibers in the VCI group were significantly different from those in the non-VCI group (*p* < 0.05). The study demonstrated that the introduction of the multi-modal CNN algorithm significantly improved the precision of MRI image segmentation and the diagnostic rate of VCI. Therefore, the multi-modal CNN algorithm based on radiomics and deep learning can be applied to the neurological assessment and disease diagnosis of VCI patients, reflecting the degree of neurological damage in patients.

Studies have shown that patients with subcortical vascular mild cognitive impairment (svMCI) and subcortical vascular dementia (SVaD) both exhibit reduced gray matter (GM) volumes, and there may be a hierarchical relationship between the two. The reduction in brain GM volume in svMCI and SVaD patients is associated with cognitive deficits [53]. However, most studies have only analyzed changes in brain structure while ignoring microstructural changes. Therefore, recent studies have also seen scholars using radiomics of the basal ganglia in MRI to diagnose and assess VCI. Therefore, Liu et al [54] used six methods, including least absolute shrinkage, to reduce the redundancy of 7106 quantitative features automatically calculated in 148 regions located in the cerebral cortex, bilateral thalamus, globus pallidus, caudate nucleus, amygdala, nucleus accumbens, putamen, and hippocampus for each subject. They employed three supervised machine learning methods—support vector machine SVM, RF, and LR—and trained diagnostic models using five-fold cross-validation, followed by evaluation of the generalization performance of each model using ten-fold cross-validation. They conducted a correlation analysis between the features of patients with subcortical ischemic vascular cognitive impairment without dementia (SIVCIND) and neuropsychological scores, identifying 13 features included in the optimal subset from the bilateral nucleus accumbens, right hippocampus, right amygdala, left thalamus, left putamen, and left caudate nucleus. Among the three models, RF achieved the best diagnostic performance, with an AUC of 0.990 and accuracy of 0.948. This study demonstrated that the combination of high-resolution T1-weighted MRI and radiomics with machine learning techniques can accurately and automatically diagnose SIVCIND, with the best radiomics features mainly located in the right amygdala, left thalamus, left caudate nucleus, and left putamen. These radiomics features may also serve as new biomarkers for SIVCIND.

Based on the studies by Xue and Liu’s teams, it is evident that radiomics and deep learning methods can be used for neurological assessment and disease diagnosis in VCI patients. This enables clinicians to better diagnose and stage VCI patients. Additionally, radiomics features primarily located in the right amygdala, left thalamus, left caudate nucleus, and left putamen can be used for radiomics analysis to more accurately diagnose SIVCIND, thereby achieving the goal of providing targeted and refined treatments. Therefore, researchers can utilize radiomics methods to diagnose VCI more quickly and conveniently.

### Application of MRI radiomics in the differential diagnosis of VCI and AD

In addition to its applications in the prediction and diagnosis of VCI, MRI radiomics is also used in the differential diagnosis of VD and AD. Since the clinical features of dementia in VD and AD are similar, it is difficult to distinguish the causes of illness when patients have both vascular disease and dementia symptoms. Some scholars have used retinal biomarkers for early diagnosis and prognosis of VD and AD [55], while others have employed a brief memory and executive function test as a cognitive screening tool to detect and differentiate VD and AD [56]. Additionally, radiomics has been utilized for the differential diagnosis of VD and AD. Zheng et al [57] explored whether multiparametric features from structural MRI could be used for the differential diagnosis of VD and AD through radiomics and machine learning. They selected 58 patients with AD and 35 patients with VD and used the AccuBrain tool to automatically segment brain tissue to extract multiparametric volumetric measurements from different brain regions. They processed a total of 62 structural MRI biomarkers and selected significant features that differed between VD and AD to reduce dimensionality. They further constructed a feature set using least absolute shrinkage and LASSO, which was then input into SVM. To fairly assess model performance, they also compared different machine learning algorithms to determine which performed best in the differential diagnosis of VD and AD and evaluated the diagnostic performance of the classification models using quantitative metrics derived from the ROC. The experimental results showed that the SVM with a radial basis function achieved good results in the differential diagnosis of VD and AD, with a sensitivity of 82.65%, specificity of 87.17%, and accuracy of 84.35% (AUC = 0.861, 95% CI = 0.820-0.902). This study utilized radiomics and machine learning to analyze patients’ brain MRI images, extract multiparametric volumetric features, and construct and evaluate diagnostic models using SVM, achieving efficient differentiation between VD and AD and providing a new approach for the diagnosis of dementia subtypes. In clinical practice, when clinicians encounter patients with both vascular disease and dementia symptoms, the application of radiomics can safely and effectively distinguish whether the dementia is caused by VD or AD, thereby ensuring precise treatment plans and improving patient outcomes.

## Limitations of MRI radiomics

MRI radiomics has shown promising results in diagnosing, predicting, and differentiating VCI from AD, but several challenges remain. Firstly, many MRI radiomics studies are retrospective with small sample sizes, often leading to a higher rate of false-positive results due to the number of extracted features exceeding the number of patients. Secondly, some studies focus on a single imaging sequence, potentially overlooking valuable contributions from other sequences. Thirdly, radiomics has yet to be widely adopted in clinical practice, requiring further validation and optimization. Moreover, the predictive value of radiomics is limited by insufficient follow-up periods in most studies. Finally, the field faces a shortage of interdisciplinary talents with expertise in both medicine and computer science, which has hindered the progress of radiomics research. Addressing these issues is crucial for advancing the clinical application and reliability of MRI radiomics.

Although MRI radiomics still has some issues, as an advanced method for quantitative analysis, radiomics has great potential for research in disease prediction and diagnosis. In the future, radiomics should become more standardized, stable, and scientific. With the rapid development of interdisciplinary medical engineering and big data in recent years, MRI radiomics will continue to advance.

## Future prospects

With the development of imaging, especially the advent of MRI, non-invasive examinations of the nervous system have been further advanced. Against the backdrop of the rapid development of interdisciplinary medical engineering, MRI radiomics has gradually come into play in neurological diseases by integrating multiple imaging data for accurate analysis, thereby reducing the risks and costs associated with biopsies or craniotomies [58]. Moreover, the emergence of MRI radiomics has brought breakthrough progress in the study of VCI. Traditional MRI examinations could not precisely predict or diagnose VCI, nor could they clearly distinguish VD from AD. However, with the development of MRI radiomics, many scholars have already achieved these goals using radiomics methods. Despite the challenges in studying VCI, such as its heterogeneous etiology, characteristics, imaging biomarkers, and complex pathological processes, the application of MRI radiomics in VCI research has enabled the analysis of nervous system function and tissue structure from multiple aspects. This has facilitated early screening of VCI by obtaining rich MRI image information and providing radiomics data and analysis as diagnostic evidence for clinicians, thereby reducing the risk of VCI for patients and offering more references for improving prognosis. In the future, precise automatic segmentation techniques [59], larger sample sizes [60], multi-sequence studies [61, 62], extended follow-up periods [63], and an increase in the number of relevant talents [64], MRI radiomics combined with clinical factors will be able to provide more accurate information for the prediction, diagnosis, and differentiation of VCI and will be successfully applied in clinical practice.

## Data Availability

The datasets used and/or analyzed during the current study are available from the corresponding author on reasonable request.

## Abbreviations

AD: Alzheimer’s disease
ADL: Activities of daily living
AUC: Area under the curve
CAA: Cerebral amyloid angiopathy
CNN: Convolutional neural networks
DCA: Decision curve analysis
GM: Gray matter
IADL: Instrumental activities of daily living
LASSO: Least absolute shrinkage and selection operator
LR: Logistic regression
LSA: Lenticulostriate artery
MCI: Mild cognitive impairment
MRI: Magnetic resonance imaging
RF: Random forest
ROC: Receiver operating characteristic
ROI: Region of interest
SIVCIND: Subcortical ischemic vascular cognitive impairment without dementia
SVaD: Subcortical vascular dementia
SVM: Support vector machines
SVMCI: Subcortical vascular mild cognitive impairment
VCI: Vascular cognitive impairment
VCIND: Vascular cognitive impairment no dementia
VD: Vascular dementia
WMH: White matter hyperintensities
